# Genome-Wide Characterization, Evolution, and Expression Profiling of *VQ* Gene Family in Response to Phytohormone Treatments and Abiotic Stress in *Eucalyptus grandis*

**DOI:** 10.3390/ijms20071765

**Published:** 2019-04-10

**Authors:** Huifang Yan, Yujiao Wang, Bing Hu, Zhenfei Qiu, Bingshan Zeng, Chunjie Fan

**Affiliations:** Key Laboratory of State Forestry Administration on Tropical Forest Research, Research Institute of Tropical Forestry, Chinese Academy of Forestry, Guangzhou 510520, China; yhuifangy@gmail.com (H.Y.); wangyujiao@caf.ac.cn (Y.W.); hubing@caf.ac.cn (B.H.); qiuzhenfei@caf.ac.cn (Z.Q.)

**Keywords:** *VQ* genes family, *Eucalyptus grandis*, expression pattern, plant hormones, abiotic stress

## Abstract

*VQ* genes play important roles in plant development, growth, and stress responses. However, little information regarding the functions of *VQ* genes is available for *Eucalyptus grandis*. In our study, genome-wide characterization and identification of *VQ* genes were performed in *E. grandis*. Results showed that 27 *VQ* genes, which divided into seven sub-families (I–VII), were found, and all but two *VQ* genes showed no intron by gene structure and conserved motif analysis. To further identify the function of EgrVQ proteins, gene expression analyses were also developed under hormone treatments (brassinosteroids, methyl jasmonate, salicylic acid, and abscisic acid) and abiotic conditions (salt stress, cold 4 °C, and heat 42 °C). The results of a quantitative real-time PCR analysis indicated that the *EgrVQs* were variously expressed under different hormone treatments and abiotic stressors. Our study provides a comprehensive overview of *VQ* genes in *E. grandis*, which will be beneficial in the molecular breeding of *E. grandis* to promote its resistance to abiotic stressors; the results also provide a basis from which to conduct further investigation into the functions of *VQ* genes in *E. grandis*.

## 1. Introduction

The VQ protein family either positively or negatively regulates diverse developmental processes, including plant immunity, abiotic stresses, and growth and development [1]. The expression of genes, which are related to biological processes and abiotic stresses in plants, is altered in response to either internal or external signals [1,2]. The *VQ* genes are a type of plant-specific proteins with a dramatically conserved VQ motif, which possesses five conserved amino acids in its main protein sequences FxxVQxhTG [3,4]. Recently, *VQ* genes have been identified by genome-wide analysis in numerous plants. There are 34, 39, 51, 29, and 74 members in Arabidopsis [5], rice [6], poplar [7], moso bamboo [8], and tea [9], respectively. Previous studies have shown that *VQ* genes played important roles in resistance to abiotic and biotic stressors [10]. Meanwhile, accumulating researches showed that many VQ proteins interacted with WRKY transcription factors [1,11]. The WRKY transcription factors, which harbor a highly conserved WRKYGQK amino acid sequence that is followed by a zinc-finger motif at the N-terminal domain, are ubiquitous among higher plants [11,12]. The VQ proteins that contain 50–60 conserved amino acids interact with WRKY transcription factors by the residues V and Q [13,14]. For example, AtVQ15 plays a negative role in response to osmotic stress [15]. AtVQ23 and AtVQ16 transcript levels were strongly induced by *Botrytis cinerea*. A study has shown that AtVQ22 negatively mediates AtWRKY28 and AtWRKY51 and it is involved with JA (jasmonic acid) defense [16]. In addition, the transcript levels of many *VQ* genes in rice differed when exposed to drought [17]. Moreover, *VQ* genes can regulate multiple biological processes including plant growth and development, senescence, and hormone signaling. The GmVQ1, GmVQ6, and GmVQ53 transcripts are highly expressed during seed development in soybean [18]. AtVQ14 and MINI3 interacted and regulated the abundance of mRNA-encoding proteins that participate in the process of seed development [16]. Specifically, *VQ* genes function by interacting with the group I or IIc WRKY transcription factors [4,19,20]. AtVQ23 and AtVQ16 activated AtWRKY33 by binding its C-terminal WRKY domain and further induced plant defenses [21]. Under salt stress, AtVQ9 expression was increased and it then interacted with WRKY8 for inhibiting the expression of its target genes to modulate salinity stress tolerance [22].

The *Eucalyptus* species are tropical/subtropical woody plants that belong to the Myrtaceae family of angiosperms. It is also the world’s leading source of woody biomass [23]. Specifically, *Eucalyptus* not only provides fuel biomass and directly reduces atmospheric carbon dioxide levels [23,24], but it also performs a variety of indirect services through its essential oils that are used as a pesticidal agent and pest repellent [25]. However, *Eucalyptus* can be affected by a variety of biotic and abiotic stressors during growth and development, including heat, cold, salt stress, and disease. *VQ* genes played a vital role in resistance to abiotic and biotic stressors. Thus, it is necessary to understand the characteristics and functions of the *VQ* genes. However, there have been no reports on *VQ* genes in *E. grandis* until now. Fortunately, the availability of the complete *E. grandis* genome provides an opportunity to conduct a comprehensive analysis of *VQ* genes in *E. grandis* [26].

In this study, *VQ* genes in *E. grandis* were identified, and then a bioinformatics analysis was conducted, including phylogenetic relationships, conserved motifs, homologous pairs, gene structures, and promoter analysis. Furthermore, the expression of *EgrVQ* genes was investigated under biotic stress conditions, such as brassinosteroids (BRs), methyl jasmonate (MeJA), salicylic acid (SA), abscisic acid (ABA) treatments, and abiotic stresses, such as cold, heat, and NaCl treatments. These results will provide a solid foundation for elucidating the function of *EgrVQ* genes in response to biotic and abiotic stress and further molecular breeding in *E. grandis*.

## 2. Results

### 2.1. Identification of the VQ Gene Family in the E. grandis

In our study, 27 *VQ* genes were identified, which contained VQ domains using *AtVQ* genes performing BLASTP search in the *E. grandis* genome and VQ conserved domain (PF05678) identification. Table 1 lists detailed information regarding the *VQ* genes in *E. grandis*. These 27 *EgrVQ* genes were named from *EgrVQ1* to *EgrVQ27*. Their translated proteins ranged from 101 to 348 amino acids (AA), with an average of 217 AAs. The predicted molecular weight of the proteins varied from 11.3 to 37.6 kDa, and the pI values ranged from 5.38 to 10.66 (Table 1). By predicting their subcellular localization, it was also found that 12 *EgrVQ* genes were located in the chloroplasts, and 14 *EgrVQ* genes were located in other compartments.

### 2.2. Mapping EgrVQ Genes on Chromosomes, Gene Duplication, and Analysis of Paralogs and Orthologs

**Figure 1:**
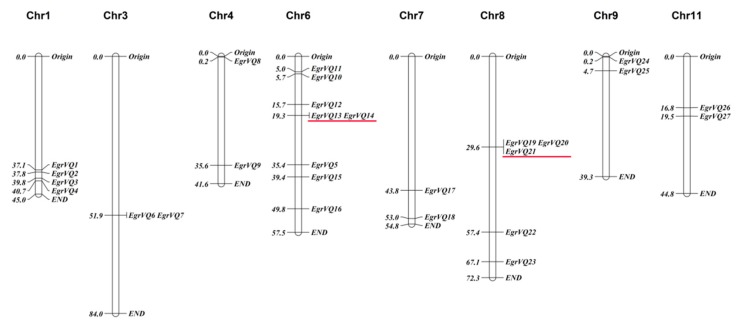
Chromosomal location of *E. grandis VQ* genes. Chromosome numbers were indicated above each chromosome. The size of a chromosome was indicated by its relative length. Gene positions and chromosome sizes were given in megabases (Mb) to the left of the figure. Tandem duplicated genes were underlined in red.

The chromosomal location illustrated that 27 *EgrVQ* genes were randomly and unequally distributed on nine chromosomes (Figure 1). Specifically, chromosome 6 contained eight *VQ* genes and chromosome 8 contained five genes, respectively. Meanwhile, the *EgrVQ13*/*EgrVQ14*, *EgrVQ19*/*EgrVQ20*, and *EgrVQ19*/*EgrVQ21*showed tandem duplication. Consistent with the gene duplication analysis, we also found that *EgrVQ13/EgrVQ14*, *EgrVQ19/EgrVQ20*, *EgrVQ19*/*EgrVQ21*, and *EgrVQ20*/*EgrVQ21* were paralogous pairs. Details from the analysis of paralogs and orthologs with *A. thaliana*, poplar, and rice are presented in Table 2.

### 2.3. Identification of Cis-Elements in the Promoter Regions of EgrVQ Genes

In this study, the identification of *cis*-regulatory elements was performed in the promoter regions of *EgrVQ* genes (Table 4). The results showed that many stress type *cis*-elements were widespread in the promoter region of *EgrVQ* genes. Table 3 shows the details information. ABA-stress, MeJA-stress, SA-stress, drought-stress, low temperature-stress, dehydration, and salt stress response elements were found in the *EgrVQs* promoter. Interestingly, all of the *EgrVQs* contained a CGTCA-motif and a TGACG-motif, which were involved in the response to MeJA. Moreover, all but *EgrVQ21* contained an ABRE element. In addition, the MBS, TCA-element, and LTR *cis*-elements, which were involved in the response to SA, drought, and low-temperature, respectively, were presented in most of the *EgrVQs* promoter regions. These results indicated that most of the *EgrVQ* genes might respond to plant biotic and abiotic stresses.

### 2.4. Multiple Sequence Alignment and Phylogenetic of EgrVQ Proteins

**Figure 2:**
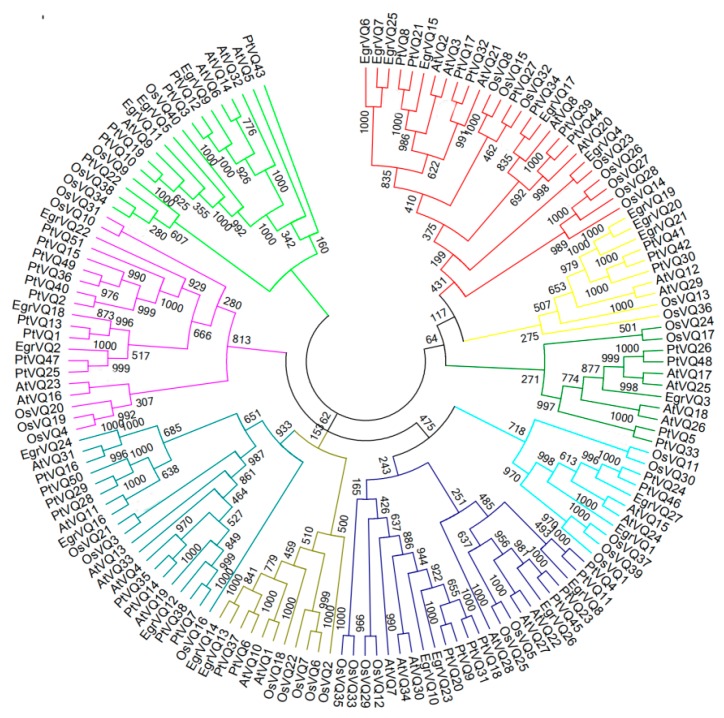
Phylogenetic tree of VQ proteins from *E. grandis*, Arabidopsis, rice, and poplar. ClustalW softwarealigned the complete amino acid sequences of 27 *E. grandis* (prefixed with ‘Egr’), 34 Arabidopsis (prefixed with ‘At’), 40 rice (prefixed with ‘Os’), and 51 (prefixed with ‘Pt’) VQ proteins, and MEGA 7, with 1000 bootstrap replicates, constructed the neighbor-joining tree.

**Figure 3:**
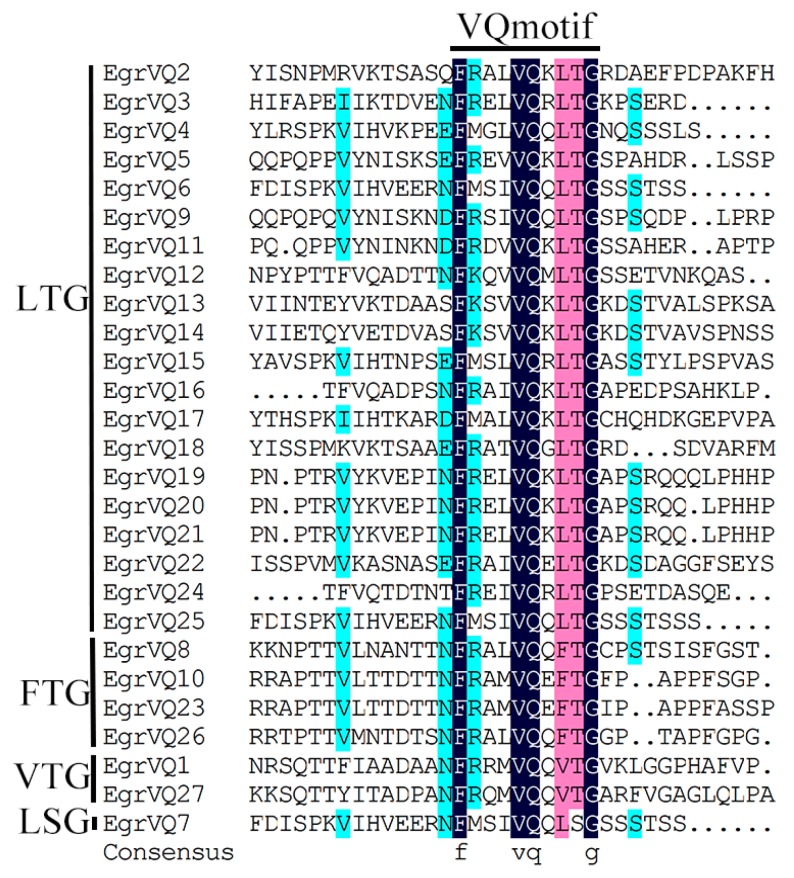
Multiple sequence alignment of VQ proteins in *E. grandis*. Sequences were aligned using DNAMAN software. The FxxxVQxxxG motif was highly conserved. The dark blue indicated the conserved section of EgrVQs protein family. The pink indicated the four conserved motif variations of LGT, FTG, VTG, and LSG.

To obtain the phylogenetic relationship of *E. grandis* VQ proteins, an unrooted tree was constructed, including 27 EgrVQ, 51 PtVQ, 34 AtVQ, and 40 OsVQ proteins (Figure 2). The results showed that EgrVQ proteins were divided into seven sub-families (I–VII). The multiple alignment analysis showed that the EgrVQ proteins presented four conserved motif variations: FxxVQxLTG(20/27), FxxVQxFTG(4/27), FxxVQxVTG(2/27), and FxxVQxLSG(1/27) (Figure 3). Among these motifs, LTG, FTG, and VTG were extensively presented in *A. thaliana* [20], rice [17], and poplar [7]. However, it was found that EgrVQ7 protein presented an LSG motif, which was not reported in previous studies. The details of the conserved motifs in *E. grandis* are presented in Table 3 and Figure 3.

### 2.5. Analysis of Gene Structural and Conserved motifs of EgrVQ Genes

**Figure 4:**
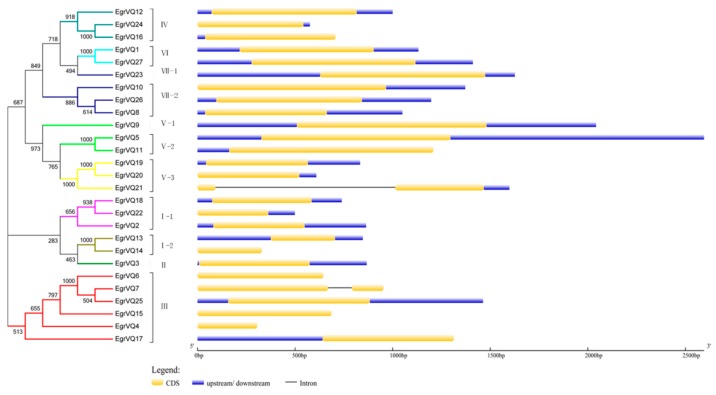
Gene structure of *VQ* genes in *E. grandis*. Exons were indicated by yellow rectangles. Upstream/downstream’s sequences of *EgrVQs* were indicated by blue lines. Gray lines connecting two exons represented introns.

**Figure 5:**
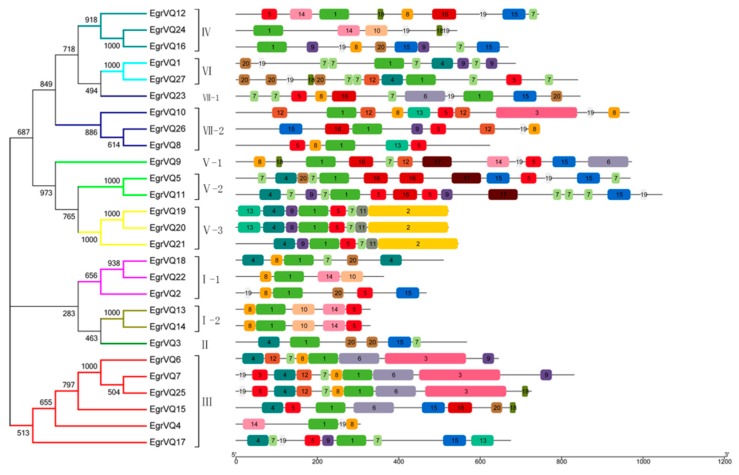
Phylogenetic relationships and conserved motifs of VQ proteins in *E. grandis*. The left panel showed the phylogenetic tree of EgrVQ, which was constructed by the neighbor-joining method based on the results of sequence alignment. Proteins were divided into seven subgroups (marked by different colors). The right panel showed the distribution of the 20 conserved motifs in the *EgrVQ* genes following analysis with Multiple Expectation Maximization for Motif Elicitation (MEME). Each specific motif was marked by a different colored box, and the motif numbers were included in the center of each box. The length of each box was proportional to the actual size of the motif.

To further understand the structural features of the *EgrVQ* genes, exon/intron structural analysis was performed and results are shown in Figure 4 and Table 1. Interestingly, 25 *EgrVQ* genes had only one exon, while the *EgrVQ7* and *EgrVQ21* genes had two exons. Subsequently, the conserved motifs of the EgrVQ proteins were studied. All of the *EgrVQ* genes contained motif1, which was the DNA-binding domain of *EgrVQ* genes (Figure 5). Notably, some motifs appeared in one sub-family of *EgrVQ*. For example, motif2, motif3, and motif17 only existed in sub-familyV-3, III, and V, respectively, which indicated that these motifs were the characteristic elements of these sub-families. Meanwhile, it was found that the same sub-family had similar motifs. For example, sub-familyV-3 genes contained motif4, motif9, motif1, motif5, motif7, and motif11, whereas sub-familyIII included motif4, motif12, motif7, motif8, motif1, motif6, and motif3. Figure 5 and Table 4 lists the details.

### 2.6. Expression Patters of EgrVQ Genes in Different Tissues of E. grandis

**Figure 6:**
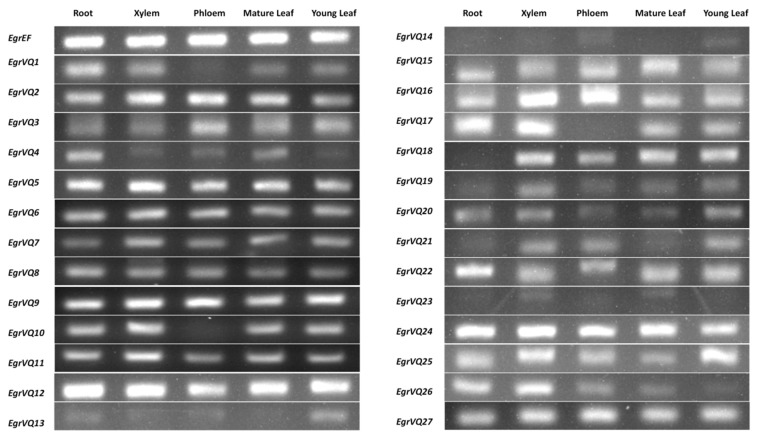
Semi-quantitative Real-Time PCR (RT-PCR) of the expression of *EgrVQ* genes in different tissues. From left to right: root, xylem, phloem, mature leaves, and young leaves. All of these tissues were collected from 6-week-old GL1 plants.

In our study, the expression patterns of *EgrVQ* genes were examined in different tissues, including root, xylem, phloem, mature leaves, and young leaves (Figure 6). The majority (88.9%) of *EgrVQ* genes were expressed in all tissues. However, almost all *EgrVQ*s showed differential gene expression. For example, from subfamily I members, *EgrVQ2* and *EgrVQ22* were relative highly expressed when compared to other members, *EgrVQ13* and *EgrVQ14* mainly were expressed in leaves, and *EgrVQ18* was not expressed in the root. Moreover, from subfamily V members, when compared to *EgrVQ20* and *EgrVQ21, EgrVQ9* and *EgrVQ11* were relative highly expressed in most tissues. In addition, from subfamily VII members, *EgrVQ1* and *EgrVQ26* were very highly expressed in root and xylem tissues than other tissues. Therefore, *EgrVQ1* and *EgrVQ26* might participate in root development and play vital roles in the xylem development of *E. grandis*.

When considering that 92.6% and 88.9% of *EgrVQ* genes were expressed in young leaves and mature leaves, respectively, we selected leaves for determining the expression profiles of *EgrVQ* genes in response to different treatments.

### 2.7. Expression Profiles of EgrVQ Genes in Response to Hormones

**Figure 7:**
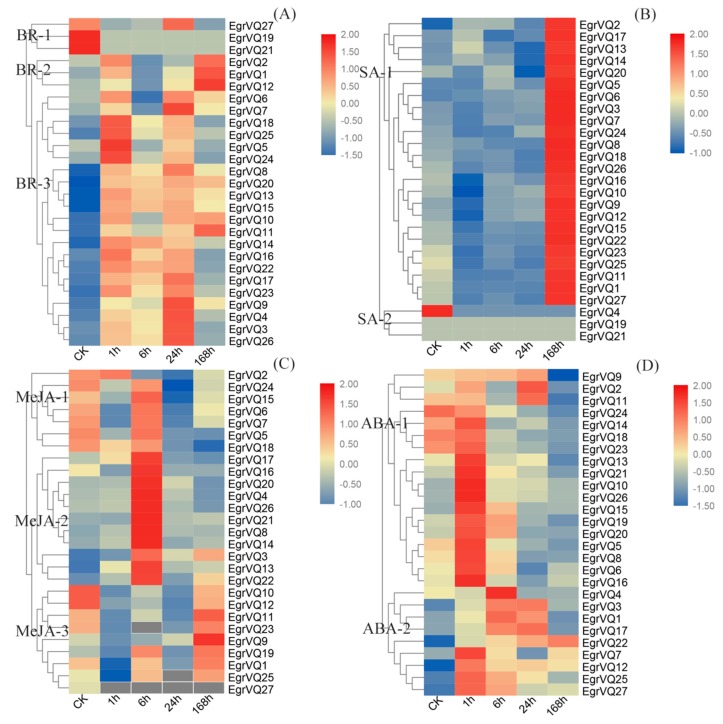
Expression analysis of 25 *EgrVQ* genes in *E. grandis* following brassinosteroid (BR) (**A**), salicylic acid (SA) (**B**), methyl jasmonate (MeJA) (**C**), and abscisic acid (ABA) (**D**) treatment, as determined by Real-Time quantitative PCR (qRT-PCR). The relative expression levels were calculated using the 2^−ΔΔCt^ method. The heatmap was created using MEV.

To investigate the function of *EgrVQ* genes in response to biotic stresses, we determined the expression patterns of *EgrVQ* genes under various plant hormone treatments, including BRs, MeJA, SA, and ABA (Figure 7). The results showed that the *EgrVQ* genes were expressed in diverse patterns under different hormone treatments. Most of the *EgrVQ* genes presented differential expression under the different phytohormone treatments. Some differences are extremely significant when compared to the reference gene *EgrEF*.

In the BR treatment, most of *EgrVQ* genes were up-regulated and the *EgrVQs* were clustered into three main groups according to their expression patterns (Figure 7A). Group BR-1 contained three *EgrVQs*, including *EgrVQ19*, *EgrVQ21*, and *EgrVQ27*, which were significantly decreased with the increasing duration of BR treatment. There were three *EgrVQs* in group BR-2 and their expression levels were dramatically increased at 1 h and 168 h after BR treatment.The rest of the *EgrVQ* genes were included in group BR-3, which contained 19 *EgrVQs* and they were continuously up-regulated.

Under SA treatment, the *EgrVQs* were mainly clustered into two groups according to their expression patterns (Figure 7B). The majority of *EgrVQs* (22/27) were clustered into group SA-1, in which the expression levels of *EgrVQs* were greatly up-regulated at 168 h, but they were not significantly altered at 1 h to 24 h after SA treatment. This result indicated that the expression of most *EgrVQs* was affected by long-term treatment with SA. The rest of the *EgrVQ*s were clustered in group SA-2 and their expression was significantly reduced or showed no obvious change.

In the MeJA treatment, the *EgrVQs* were mainly clustered into three groups according to their expression patterns (Figure 7C). In the MeJA-1 group, there were seven *EgrVQs*, and their expressions were greatly decreased at 1 h, 24 h, and 168 h after MeJA treatment. The MeJA-2 group contained eleven *EgrVQs*, which showed significantly decreased expression at 1 h and then increased the expression at 6 h and reached their maximum expression level and decreased afterward. The rest of *EgrVQ*s were clustered in group MeJA-3, in which the expression levels of *EgrVQs* were greatly down-regulated at 1–24 h, and increased back to the level of the control or increased. However, it was found that *EgrVQ*s were immediately down-regulated under MeJA treatment.

Under ABA treatment, the expression level of the majority of *EgrVQs* was obviously reduced at 168 h and the *EgrVQs* were mainly clustered into two groups (Figure 7D). In group ABA-1, the expression of *EgrVQs* was significantly up-regulated at 1 h and then decreased with increasing treatment time under ABA treatment. In group ABA-2, there were nine *EgrVQs*, and their expression levels increased at 1 h, 6 h, or 24 h.

### 2.8. Expression Profiles of EgrVQ Genes in Response to Stress Treatments

**Figure 8:**
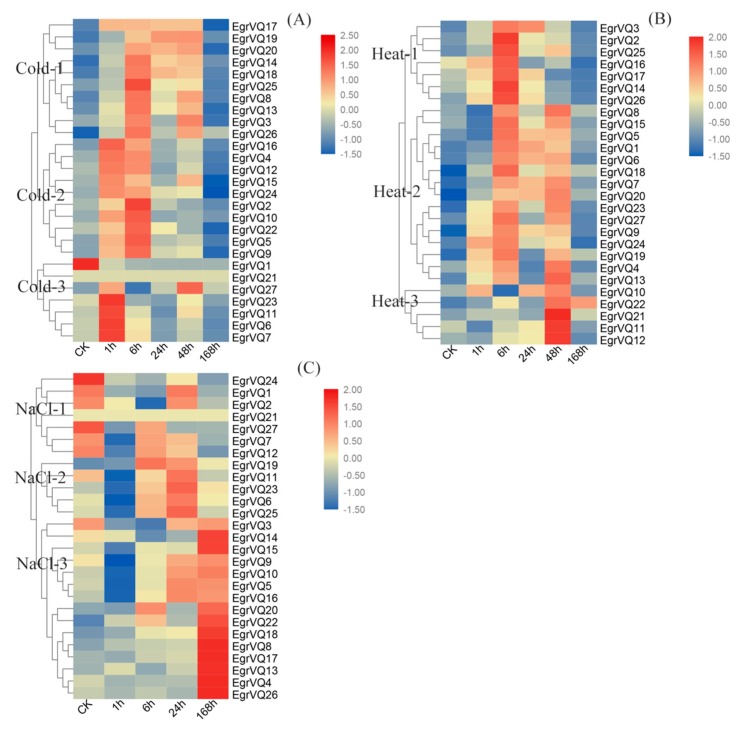
Expression analysis of 25 *EgrVQ* genes in *E. grandis* following cold (**A**), heat (**B**), and NaCl (**C**) treatments, as determined by qRT-PCR. The relative expression levels were calculated using the 2^−ΔΔCt^ method. The heatmap was created using MEV.

To research the function of *EgrVQ* genes in response to abiotic stressors, we investigated the expression patterns of *EgrVQ* genes under NaCl, cold, and heat treatments, respectively (Figure 8). Overall, the *EgrVQ* genes showed differential expression under the different abiotic stresses. Some differences are even significant when compared to the reference gene *EgrEF*. Specifically, most of *EgrVQ* genes were up-regulated at 1 h or 6 h and they reached their highest expression levels, but they were then down-regulated at 168 h. These results indicated that *EgrVQ* genes could respond to short-term temperature stress. Interestingly, it was found that similar expression patterns of *EgrVQ* genes occurred under cold and heat treatments.Subsequently, *EgrVQs* were classified into three groups both in the cold and heat treatments, respectively.

In group Cold-1 and group Heat-2, there were 10 and 15 *EgrVQs*, respectively. The expression patterns of these genes were similar, and their expressions were greatly increased at 6-48 h and then decreased after ABA treatment. Similarly, 10 and seven *EgrVQs* were classified into group Cold-2 and group Heat-1, respectively. Their expressions levels were greatly increased at 1 h and 6 h, but they then decreased after ABA treatment. In group Cold-3, *EgrVQs* were significantly increased at 1 h. In group Heat-3, *EgrVQs* were significantly increased at 48 h.

Under NaCl treatment, the *EgrVQs* were clustered into three groups according to their expression patterns (Figure 8C). In group NaCl-1, the expression of *EgrVQs* showed slight decreases with an increasing treatment time. In group NaCl-2, *EgrVQ*s were down-regulated at 1 h and then up-regulated with increasing NaCl treatment duration at 6 h and 24 h. The remaining of *EgrVQs* were classified to group NaCl-3. In this group, the expression of *EgrVQs* gradually increased and reached their highest expression level at 168 h.

## 3. Discussion

In our study, 27 *EgrVQ* genes were identified using the genome database in *E. grandis*. Subsequently, phylogenetic analysis, conserved motifs, and analysis of *cis*-elements of *EgrVQs* were performed. All of the *EgrVQs* encoded relatively small proteins of less than 400 amino acids, and most VQ motif-containing proteins contained FxxVQxLTG(20/27), FxxVQxFTG(4/27), and FxxVQxVTG(2/27). Moreover, most *EgrVQs*(25/27) only contained an exon. Our results were consistent with *A. thaliana*, poplar, and rice [7,17,20], which indicated that *VQ* genes were relatively conservative. However, motif FxxVQxLSG(1/27), which was not shown in *A. thaliana*, rice, poplar, or grape, was found in *E. grandis* (Table 3).

Additionally, according to 20 conserved motifs in the analysis of *EgrVQ* genes with Multiple Expectation Maximization for Motif Elicitation (MEME), motif1 was presented in all of the *EgrVQ* genes (Figure 5). This result was consistent with other plants, like poplar [7] and moso bamboo [8,27], implying that the motif 1 imparts specific functions to the VQ protein. Moreover, it was found that several *cis*-elements that related to plant hormones and abiotic stressors were identified in the promoter region of the *EgrVQ* genes (Table 4). Similarly, most of these *cis*-elements occurred in other plants, like rice [17], poplar [7], and bamboo [8,27]. Interestingly, DRE is a *cis*-acting element that is involved in salt stress,cold stress, and dehydration, which was only presented in *EgrVQ20* and *EgrVQ21* (Table 4), and also seldom occurred in other plants.

In a previous study, VQ proteins participated in regulating diverse developmental processes, especially in response to biotic and abiotic stressors [20]. SA and JA are vital defense signaling molecules to response to pathogen infection and other abiotic stress conditions. In our study, the transcription of most *EgrVQ* genes was altered under SA and MeJA treatments. Interestingly, the expression of many *EgrVQ* genes was significantly increased at 168 h of SA treatment, which was not studied in moso bamboo [27], *A. thaliana* [20], and *Vitisvinifera* [28]. It is worth noting that *EgrVQ2*, *EgrVQ18*, and *EgrVQ22*, which were homologs of *AtVQ23* and *AtVQ16*, were highly expressed at 168 h under SA treatment. In *A. thaliana*, the over-expression of *AtVQ23* induced hyper-activate defense-related genes in plants following pathogen infection or SA and MeJA treatments, which enhanced resistance to infection by *Pseudomonassyringae* [29]. Meanwhile, *AtVQ16* could regulate the immune response by regulating *AtWRKY33* and further stimulating the DNA binding activity [30]. It was also found that *EgrVQ2*2 expression also increased under MeJA treatment. However, *EgrVQ2* expression was not significantly changed and *EgrVQ18* expression was slightly down-regulated under MeJA treatment. Hence, these results implied that *EgrVQ18* could respond to SA treatment and it was further involved in SA-mediated defense responses by the long-term effect. *EgrVQ22* could also respond to SA and MeJA treatments.

Moreover, *AtVQ22*, which was positively regulated by the COI1 (CORONATINEINSENSITIVE1)-dependent signaling pathway, was a master controller regulating JA-mediated plant response against insects and pathogens [16,31]. *EgrVQ8*, which was homologous with *AtVQ22*, was induced at 6 h under MeJA treatment and 168 h under SA treatment, indicating that *EgrVQ8* has a similar function in *E. grandis.* However, *EgrVQ26*, which is another homologous gene with *AtVQ22*, was not changed under MeJA treatment.

Furthermore, ABA and BR were also important in the response of plants to abiotic stressors [32,33]. In our study, most *EgrVQs* were up-regulated at 1 h–24 h when the plants were exposed to ABA, which was similar to moso bamboo *VQ* genes [8]. Meanwhile, only three genes showed up-regulation and multiple *OsVQ* genes showed down-regulation after 12 h under ABA treatment [17]. However, we found that 22 *EgrVQs* showed significantly down-regulation at 168 h under ABA treatment. These results indicated that the VQ genes had special roles in response to ABA treatment in *E. grandis*. It was also found that *EgrVQ* subfamily I members, including *EgrVQ2*, *EgrVQ13*, *EgrVQ14*, *EgrVQ22*,and *EgrVQ18*, were highly expressed under ABA treatment,which was similar with *OsVQ6*, *OsVQ7*, and *OsVQ10* belonging to subfamily. These results implied that these genes have similar functions and they might be related to the ABA signaling pathway. Specifically, *AtVQ15* regulated the osmotic stress tolerance with *WRKY25* and *WRKY51* in *A. thaliana* [20]. Its homologous gene, *EgrVQ27*, was significantly up-regulated after the ABA treatment, suggesting that *EgrVQ27* might affect osmotic stress tolerance in *E. grandis*. In addition, we found that most of the *EgrVQ* genes were highly expressed under the BR treatment (Figure 7A). BRASSINAZOLE-RESISTANT 1(BZR1), which is a key transcription factor (TF) in the BRs signal pathway, can trigger the expression of *FLS2* and *SNC1* to enhance pathogen resistance in plants [34,35].These results implied that *EgrVQ* genes might take part in enhancing the pathogen resistance of the BR signal pathway.

Plants also need to evolve efficient defense systems to protect themselves from abiotic stressors, like extreme temperature, salt, and so on. AtVQ9 acted as a repressor of WRKY8 to maintain a balance in the activation of WRKY8-mediated signaling pathways that are involved in salt stress in *A. thaliana* [22]. In our study, *EgrVQ5* and *EgrVQ9*, both homologous of *AtVQ9*, showed the same expression trends and they were increased at 168 h under NaCl treatment (Figure 8C). This indicated that the *VQ* genes were relatively conservative and were also involved in salt stress. In contrast, another homologous gene, *EgrVQ11*, was decreased at 168 h. *AtVQ15* was induced by dehydration and high salinity, whereas lines that overexpressed this gene showed an increased desensitivity to salt stress during seed germination and seedling growth [20,22]. Interestingly, its homologous gene, *EgrVQ1*, showed obviously decreased expression under NaCl treatment. We speculate that there is a difference in the woody and herbaceous plant.

Furthermore, few previous studies have been conducted on the function of *VQ* genes under heat and cold stresses in the woody plants. Our results showed that 23 and 24 *EgrVQ* genes were up-regulated at 1 h or 6 h, respectively, and then down-regulated at 168 h under cold and heat treatments, respectively (Figure 8A,B), which was consistent with Chinese Cabbage (*Brassica pekinensis*) [36]. It is noteworthy that most of the *EgrVQ* genes showed similar expression patterns between heat and cold treatment, suggesting that *EgrVQ*s existed in the same response pathway to heat and cold stress.

Overall, our results implied various stressors motivated or repressed a batch of *EgrVQ*s. These results might aid in the selection of available candidate genes in the *EgrVQ* gene family for deeper functional characterization.

## 4. Materials and Methods

### 4.1. Identification of VQ Gene Family in E. grandis

We conducted a systematic search on related bioinformatics analysis websites in order to acquire all of the proteins in *Eucalyptus*. Firstly, the sequences of *VQ* genes of *A. thaliana*, rice, and poplar were downloaded. Afterwards, the *VQ* genes were searched in *E. grandis* was using in NCBI (https://www.ncbi.nlm.nih.gov/), UniProt (https://www.uniprot.org), and EucGenIE (https://eucgenie.org/) databases. Next, all of the predicted *VQ* genes were subjected to SMART (http://smart.emblheidelberg.de/) and Pfam (https://pfam.sanger.ac.uk/) to confirm that they contained the VQ motif (PF05678). Lastly, physical parameters of *VQ* genes, including open reading frame (ORF) length, protein length, isoelectric point (pI), and molecular weight were calculated in ExPASy (http://www.expasy.org/tools) [37]. Subcellular localization was predicted using the WoLF PSORT (http://wolfpsort.org/) [38] and TargetP 1.1 (http://www.cbs.dtu.dk/services/TargetP/) servers [39].

### 4.2. Chromosomal Location, Gene Duplication, and Identification of Paralogs and Orthologs of EgrVQ Genes

MapChart 2.30 software drew the picture of chromosomal location [40] on the basis of initial position information that was provided in Phytozomev12.1.6 (https://phytozome.jgi.doe.gov/pz/portal.html#). In addition, *EgrVQ* genes, which are located on duplicated chromosomal blocks, were considered to undergo segmental duplication [26]. Next, we used a previously described method for the analysis of paralogs and orthologs [41]. For the elected species, all-against-all nucleotide sequence similarity searches were done among the protein sequences by BLASTN software [42]. Specifically, in one species, a pair of matching sequences was shown as pairs of paralogs, which, when aligned, exceed 300bp and the identity was over 40% [7]. In two different species, the two sequences were defined as orthologs with the reciprocal best hits for each being within >300bp of the aligned sequences from two different species [7,8].

### 4.3. Promoter Analysis of EgrVQ Genes

The 2,000-bp upstream sequences of the transcriptional start sites of *EgrVQ*genes were submitted to PlantCARE (http://bioinformatics.psb.ugent.be/webtools/plantcare/html/) [43] to identify the putative *cis*-elements.

### 4.4. Phylogenetic Analysis and Multiple Sequence Alignment of EgrVQ Proteins

Multiple sequence alignment of 27 VQ full-length protein sequences from *Eucalyptus* was performed using ClustalX2.11 [44]. A phylogenetic tree was constructed with the neighbor-joining (NJ) method in MEGA 7 (https://www.megasoftware.net/home) [45], with 1,000 bootstrap replicates. All VQ protein sequences from *A. thaliana*, poplarand rice were conducted using ClustalX2.11 and MEGA7 software.

### 4.5. Gene Structure and Conserved Motifs Analysis of EgrVQ Genes

The exon-intron structures of the *EgrVQ* genes were investigated using Gene Structure Display Server (GSDS: http://gsds.cbi.pku.edu.ch) [46] by aligning cDNA to their corresponding genomic region. In addition, the Multiple Expectation Maximization for Motif Elicitation (MEME) program (http://meme-suite.org/tools/meme) [47] was used to identify the conserved motifs. The parameters were set, as follows: the optimum motif width ranged from 6 to 200, the maximum number of motifs was 20, and other parameters were set at default.

### 4.6. Plant Material and Treatments

We used approximately 20 cm tall, six-week-old *E. grandis* GL1 clones in hydroponics conditions. The trees were grown in the greenhouse of the Research Institute of Tropical Forestry, Chinese Academy of Forestry, Guangzhou, China.

Hormone treatments were carried out by spraying the leaves of individual plants with 100 nM Epi-Brassinosteroid (Epi-BR) and 100 μΜ ABA, SA, and MeJA, respectively. For the salt stress treatment, the *E. grandis* plants were transferred to 200 mM NaCl solution and cultured for a total of 168 h; leaves were collected after 0, 1, 6, 24, and 168 h. For cold and heat stress treatments, the plants were transferred into 4 °C and 42 °C growth chamber. Then leaves were collected after 0, 1, 6, 24, 48, and 168 h of treatment. All of the samples were immediately frozen in liquid nitrogen and stored at −80 °C for subsequent total RNA extraction. The leaves from at least three plants were collected, and all treatments were performed in triplicate.

### 4.7. RNA Extraction, Semi-Quantitative Real-Time PCR (RT-PCR), and Real-Time Quantitative PCR (qRT-PCR)

Total RNA was extracted using Aidlab plant RNA kit (Aidlab Biotech, Beijing, China). The concentration and qualification were checked by NanoDrop™ One/OneC (ThermoFisher SCIENTIFIC, Waltham, MA, USA). Total RNA (1.0 μg) was used for first-strand cDNA synthesis using SuperScriptIII (Invitrogen), following the manufacturer’s instructions. Gene-specific primers (Supplement Table S1) were designed by using Premier 5.0. qRT-PCR was performed on the Roche LightCycle 96 by using TB Green *Premix Ex Taq*II (TliRNaseH Plus) (RR420Q TaKaRa Biotechnology, Beijing, China), with a total sample volumeof 20 μL. The programs were 95 °C for 30 s, then 40 cycles of 95 °C for 5 s, and 65 °C for 34 s. The relative expression level was calculated while using the 2^−ΔΔCT^ method [48]. The *EgrEF* was used as the reference gene [49].

### 4.8. Statistical Analysis

Statistical analysis was performed using OFFICE Excel and SPSS. The mean values and standard deviations (SDs) were obtained from three biological and three technical replicates; statistical significance was showed by one-way ANOVA, LSD (Least-Significant Difference).

## 5. Conclusions

Recently, an increasing number of *VQ* genes in different plants were analyzed in genome-wide characterization and evolutionary studies. However, there is little functional information regarding *VQ* genes in the woody plants. Our research showed an overall picture of *EgrVQ* genes, displaying the genome-wide identification, characterization, and expression of *EgrVQ* genes under various stressors. The results verified that the *EgrVQ* genes family either positively or negatively regulated multiple responses, including biotic and abiotic stressors. In addition, this study will provide a theoretical basis for further study of the mechanism of the stress resistance mechanism of the *VQ* gene family and indicated that most of the *VQ* genes participate between the process of biotic and abiotic stress resistance of *E. grandis*.

## Figures and Tables

**Table 1:** The summary of 27 identified *EgrVQ* genes.

Name	Gene ID	Chr	Location Coordinates (5′–3′)	ORF Length (bp)	Exons	Protein Length (aa)	Protein MW (Da)	Protein PI	Subcellular WoLF PSORT	Localization TargetP
*EgrVQ1*	Eucgr.A02182.1	1	37113647..37114778	687	1	228	22929.82	9.50	Nucleus	Chloroplast
*EgrVQ2*	Eucgr.A02242.1	1	37821284..37822147	468	1	155	16913.17	7.92	Mitochondrion	Chloroplast
*EgrVQ3*	Eucgr.A02450.1	1	39836460..39837326	567	1	188	21252.59	5.38	Nucleus	Any other location
*EgrVQ4*	Eucgr.A02544.1	1	40671118..40671423	306	1	101	11278.94	9.36	Cytoplasm	Chloroplast
*EgrVQ5*	Eucgr.B00035.1	6	35375963..35378557	969	1	322	34040.85	10.02	Nucleus	Chloroplast
*EgrVQ6*	Eucgr.C02621.1	3	51875030..51875674	645	1	215	22454.41	6.65	Mitochondrion	Chloroplast
*EgrVQ7*	Eucgr.C02629.1	3	51927510..51928462	831	2	276	28709.31	7.15	Nucleus	Chloroplast
*EgrVQ8*	Eucgr.D00026.1	4	216578..217627	62	1	207	22664.50	5.91	Nucleus	Any other location
*EgrVQ9*	Eucgr.D02200.1	4	35568137..35570178	972	1	323	35209.97	10.66	Nucleus	Any other location
*EgrVQ10*	Eucgr.F00302.1	6	5689343..5690714	966	1	321	34599.00	9.66	Nucleus	Chloroplast
*EgrVQ11*	Eucgr.F00371.1	6	5040555..5041763	1047	1	348	37574.61	9.99	Nucleus	Chloroplast
*EgrVQ12*	Eucgr.F01148.1	6	15726039..15727038	744	1	247	26541.68	9.01	Nucleus	Chloroplast
*EgrVQ13*	Eucgr.F01407.1	6	19292825..19293671	330	1	109	12303.87	7.79	Chloroplast	Any other location
*EgrVQ14*	Eucgr.F01408.1	6	19298931..19299260	330	1	109	12161.73	6.73	Cytoplasm	Any other location
*EgrVQ15*	Eucgr.F02723.1	6	39354381..39355067	687	1	228	24468.88	9.47	Nucleus	Any other location
*EgrVQ16*	Eucgr.F03907.1	6	49847862..49848569	669	1	222	23853.03	9.71	Nucleus	Any other location
*EgrVQ17*	Eucgr.G02312.1	7	43757342..43758655	675	1	224	24656.69	8.68	Nucleus	Chloroplast
*EgrVQ18*	Eucgr.G03218.1	7	52999148..52999886	510	1	169	17744.58	6.50	Nucleus	Any other location
*EgrVQ19*	Eucgr.H02317.1	8	29626932..29627764	522	1	173	18910.74	9.93	Chloroplast	Any other location
*EgrVQ20*	Eucgr.H02318.1	8	29632300..29632908	522	1	173	18883.63	9.93	Chloroplast	Any other location
*EgrVQ21*	Eucgr.H02319.1	8	29636679..29638276	546	2	181	19910.03	10.05	Chloroplast	Secretory pathway
*EgrVQ22*	Eucgr.H04261.1	8	57364319..57364817	363	1	120	13296.04	8.87	Nucleus	Chloroplast
*EgrVQ23*	Eucgr.H04807.1	8	67074212..67075837	846	1	281	29271.01	8.93	Nucleus	Any other location
*EgrVQ24*	Eucgr.I00016.1	9	223638..224213	543	1	180	19892.34	9.00	Nucleus	Any other location
*EgrVQ25*	Eucgr.I00226.1	9	4661485..4662947	726	1	241	25294.49	6.29	Nucleus	Chloroplast
*EgrVQ26*	Eucgr.K01342.1	11	16771278..16772474	747	1	248	25715.14	10.08	Nucleus	Any other location
*EgrVQ27*	Eucgr.K01686.1	11	19466093..19467503	840	1	279	28773.80	6.59	Nucleus	Any other location

**Table 2:** The summary of paralogous (*Egr–Egr*) and orthologous (*Egr–At*, *Egr–Os* and *Egr-Pt*) gene pairs.

*Egr–Egr*	*Egr–At*	*Egr–Os*	*Egr-Pt*
*EgrVQ6/EgrVQ7*	*EgrVQ1/AtVQ24*	*EgrVQ12/OsVQ3*	*EgrVQ9/PtVQ3*
*EgrVQ6/EgrVQ25*	*EgrVQ5/AtVQ9*	*EgrVQ12/OsVQ21*	*EgrVQ9/PtVQ12*
*EgrVQ19/EgrVQ20*	*EgrVQ9/AtVQ6*	*EgrVQ15/OsVQ8*	*EgrVQ12/PtVQ7*
*EgrVQ19/EgrVQ21*	*EgrVQ11/AtVQ9*	*EgrVQ15/OsVQ15*	
*EgrVQ20/EgrVQ21*	*EgrVQ12/AtVQ4*	*EgrVQ24/OsVQ3*	
	*EgrVQ12/AtVQ19*		
	*EgrVQ12/AtVQ33*		
	*EgrVQ13/AtVQ1*		
	*EgrVQ13/AtVQ10*		
	*EgrVQ14/AtVQ1*		
	*EgrVQ14/AtVQ10*		
	*EgrVQ15/AtVQ3*		
	*EgrVQ15/AtVQ21*		
	*EgrVQ16/AtVQ11*		
	*EgrVQ24AtVQ31*		

**Table 3:** The number of structural analysis of conserved motif FxxVQxxxG in *E. grandis* and other plants.

Gene Name	LTG	FTG	VTG	LTS	LTD	YTG	ITG	LTR	LTV	ATG	LTA	LSG
Arabidopsis	24	5	2	1	1	1	0	0	0	0	0	0
Poplar	39	11	2	0	0	0	0	0	0	0	0	0
Chinese Cabbage	43	8	3	1	0	1	0	0	1	0	0	0
Soybean	55	15	2	1	0	1	0	1	0	0	0	0
Grapevine	14	3	1	0	0	1	0	0	0	0	0	0
Maize	42	8	6	0	0	0	2	0	0	2	1	0
Rice	28	7	4	0	0	1	1	0	0	0	0	0
*Eucalyptus grandis*	20	4	2	0	0	0	0	0	0	0	0	1

**Table 4:** Summary of stresses inducible *cis*-elements in the promoter regions of *VQ* genes in *E. grandis*.

Gene Name	ABRE ^1^	CGTCA-motif ^2^	TGACG-motif ^3^	TCA-element ^4^	MBS ^5^	LTR ^6^	DRE ^7^
*EgrVQ1*	1	1	1		1	1	
*EgrVQ2*	1	1	1	1	1		
*EgrVQ3*	1	1	1		1		
*EgrVQ4*	1	1	1		1	1	
*EgrVQ5*	1	1	1			1	
*EgrVQ6*	1	1	1		1		
*EgrVQ7*	1	1	1			1	
*EgrVQ8*	1	1	1	1			
*EgrVQ9*	1	1	1	1		1	
*EgrVQ10*	1	1	1	1	1	1	
*EgrVQ11*	1	1	1	1			
*EgrVQ12*	1	1	1				
*EgrVQ13*	1	1	1			1	
*EgrVQ14*	1	1	1			1	
*EgrVQ15*	1	1	1		1	1	
*EgrVQ16*	1	1	1	1		1	
*EgrVQ17*	1	1	1			1	
*EgrVQ18*	1	1	1		1	1	
*EgrVQ19*	2	1	1		1	1	
*EgrVQ20*	1	1	1				1
*EgrVQ21*		1	1	1			1
*EgrVQ22*	1	1	1		1		
*EgrVQ23*	1	1	1	1		1	
*EgrVQ24*	1	1	1	1			
*EgrVQ25*	1	1	1	1	1		
*EgrVQ26*	1	1	1	1		1	
*EgrVQ27*	1	1	1	1	1		

^1^ cis-acting element involved in the abscisic acid responsiveness; ^2^ cis-acting regulatory element involved in the MeJA-responsiveness; ^3^ cis-acting regulatory element involved in the MeJA-responsiveness; ^4^ cis-acting element involved in salicylic acid responsiveness; ^5^ MYB binding site involved in drought-inducibility; ^6^ cis-acting element involved in low-temperature responsiveness; ^7^ cis-acting element involved in dehydration, low-temperature, and salt stresses.

## Supplementary Materials

Supplementary materials can be found at https://www.mdpi.com/1422-0067/20/7/1765/s1.
